# Prevalence and tick loads in Nguni cattle reared in different environmental conditions across four provinces of South Africa

**DOI:** 10.14202/vetworld.2022.1943-1953

**Published:** 2022-08-17

**Authors:** Ntanganedzeni O. Mapholi, C. Banga, K. Dzama, O. Matika, V. Riggio, N. Nyangiwe, A. Maiwashe

**Affiliations:** 1 Department of Agriculture and Animal Health, University of South Africa, Florida, South Africa; 2 Botswana University of Agriculture and Natural Resources, Gaborone, Botswana; 3 Department of Animal Sciences, University of Stellenbosch, Matieland, South Africa; 4 The Roslin Institute and R(D)SVS, University of Edinburgh, Edinburgh, United Kingdom; 5 Centre for Tropical Livestock Genetics and Health, Roslin Institute, University of Edinburgh, Edinburgh, United Kingdom; 6 Dohne Agricultural Development Institute, Eastern Cape Province, South Africa; 7 Agricultural Research Council, Irene, South Africa

**Keywords:** *Amblyomma hebraeum*, Nguni cattle, South Africa, tick count, tick prevalences, warmer climate

## Abstract

**Background and Aim:**

In tropical and subtropical countries, ixodid ticks are among livestock’s most economically important ectoparasites. Although Nguni cattle from South Africa have adapted to harsh environments, it is unknown whether they will be resistant to ticks, and the diseases carried by ticks under various climatic conditions. Therefore, this study aimed to compare tick load and estimate the prevalence of different tick species among Nguni cattle under different environmental conditions.

**Materials and Methods:**

Tick counts were conducted monthly under natural challenges over 2 years on 586 Nguni cattle located at ARC-Roodeplaat and Loskop farms (warmer climate), Mukhuthali Nguni Community and the University of Fort Hare farms (cooler climate). The generalized linear model procedure of the Statistical Analysis System was used to analyze the data. It fitted the location (farm), sex, year, month or season, and animal age as covariates.

**Results:**

The tick species (relative prevalence) observed were as follows: *Amblyomma hebraeum* (42%), *Rhipicephalus evertsi* (22%), *Rhipicephalus* (*Boophilus*) spp. (16%), *Rhipicephalus appendiculatus* (11%), *Hyalomma marginatum* (5%), and *Rhipicephalus simus* (4%). Tick infestation was significantly affected by location, season, year, month of the tick counting and age of the animal. Loskop farm had the highest tick count (m = 30.69) and showed the largest variation in tick count. Compared to the other seasons, higher tick counts were seen during the hot-dry (September–November) and hot-wet (December–February) seasons. *A. hebraeum* was the dominant tick species across all four farms, followed by *R. evertsi*. The perianal region (under the tail head), the perineum and the belly body locations were the most preferred tick attachment sites.

**Conclusion:**

These results provide useful information for developing appropriate control strategies for ticks and tick-borne diseases in these provinces of South Africa. Further work must investigate the feasibility of genetic improvement for tick resistance.

## Introduction

Global warming has led to a growing interest in farming with cattle adapted to challenging environments, especially in Africa’s tropical and subtropical regions [1]. The high incidence of diseases and parasites in these environments results in large economic losses [2, 3]. Thus, farmers must use animals well adapted to these environmental conditions to maximize production efficiency. In livestock production, ticks are among the most economically significant ectoparasites and disease pathogen vectors [4–6]. Livestock production in South Africa is heavily affected by ten Ixodid ticks [7–9]. The most economically important tick genera affecting cattle production in South Africa are *Rhipicephalus* (including the genus formerly known as *Boophilus*), *Amblyomma* and *Hyalomma* [9–12]. These tick genera impact animal productivity directly through heavy infestation or indirectly by transmitting tick-borne diseases (TBDs) [10, 13]. They transmit diseases and produce toxins, and the most important TBDs affecting cattle production in South Africa are ehrlichiosis (heartwater), babesiosis (redwater), anaplasmosis (gallsickness), and theileriosis (corridor disease). In addition to reducing weight gain, milk yield, and inflicting tick bites which result in wounds and infections, ticks also impact production [9, 14, 15]. Tick bites from the long mouth part of the ticks leave scars on the skin, which causes depreciation in the quality and price of leather products. Control strategies such as chemical control, tick vaccination and grazing management to eradicate ticks have been used; however, these strategies may only control ticks temporarily [16].

It has been suggested that if global warming leads to temperature increases, the abundance of ticks will increase in some regions where ticks are endemic [17]. This could have negative effects on tick infestation, the prevalence of TBDs and tick-borne disease prevention methods in the tropical environment. Agroecological conditions, seasonal variations and host species differences influence tick infestation [18–20]. Therefore, climatic factors such as temperature, precipitation and humidity impact tick populations, especially for tick species that prefer warmer, humid climates. Evidence can be seen in the displacement of *Rhipicephalus decoloratus* by *Rhipicephalus microplus* in Africa, which has been associated with changes in climatic conditions and cattle movement [12, 21–26], and *Amblyomma hebraeum* distribution is escalating in the inland semi-­arid areas of South Africa. It has also been associated with intense periods of drought, especially in the inland highlands [27, 28]. Finding effective control strategies for these increasing tick infestations has become a concern in the livestock industry due to these findings.

Genetic variation in tick resistance, within and between breeds, exists and is well recognized [29, 30]. Several exotic cattle breeds in South Africa are susceptible to ticks and TBDs [31]. Most of these breeds have a high production potential. However, production is compromised by TBDs. Indigenous breeds and some locally developed breeds in South Africa have adapted to harsh tropical conditions. Nguni cattle are known for their adaptation to tropical and semi-arid regions of the Southern African region and are extensively used by commercial and emerging farmers [31–33]. Their genetic potential to tick resistance has been reported as one of the key tools in establishing cattle breeds that are tick resistant [34, 35]. Spickett *et al*. [9] reported that Nguni cattle are more resistant to natural tick infestation than Bonsmara and Hereford cattle. Rechav *et al*. [36] reported that Nguni cattle were more resistant to infestation by *R. decoloratus* than five other breeds of cattle considered in their study. Moreover, Marufu *et al*. [37] noted that Nguni cattle are more tick-resistant than crossbred cattle when grazing on rangeland.

Although Nguni cattle carry lower tick loads than crossbred and exotic cattle, little is known about the variation in tick loads within this locally adapted breed. Furthermore, climate change is expected to induce differences in the prevalence of different tick species [1]. Therefore, tick loads and the prevalence of ticks must be assessed at animal and species levels in order to better understand the implications of environmental change on livestock production. Knowledge of agroclimatic and animal factors influencing tick load and prevalence in Nguni cattle is also important.

Thus, this study assessed tick loads and estimated the prevalence of different species of ticks in Nguni cattle reared in different environmental conditions in South Africa. This study did not explore the molecular mechanism involved under different loads and the prevalence of ticks in Nguni cattle. However, the knowledge gained from this study offers fundamental tools for developing strategies to control tick infestation in cattle under various agroclimatic conditions.

## Materials and Methods

### Ethical approval

The Agricultural Research Council and Eastern Cape Department of Agriculture gave written consent for their respective research farms to participate in the study. The study was approved by the Agricultural Research Council, Animal Production Institute Ethics Committee (Ref: APIEC15/012).

### Study period and location

The study was conducted from May 2012 to April 2014. Tick counts were conducted on 586 Nguni cattle from four locations, Limpopo Province’s Loskop Research Farm of the Agricultural Research Council, ARC-Roodeplaat Research Farm is located in Gauteng Province, Mukhuthali Nguni Community Farm is located in the Kwa-Zulu Natal Province and The University of Fort Hare farm is located in Alice in the Eastern Cape Province, all from South Africa.

### Experimental cattle and sampling areas

Nguni cattle of both sexes were randomly sampled from four different herds in different provinces of South Africa. Each selected herd was managed on its original farms. The age of cattle and their physiological status varied in each location. Some of the herds had available pedigree information.

**Figure-1:**
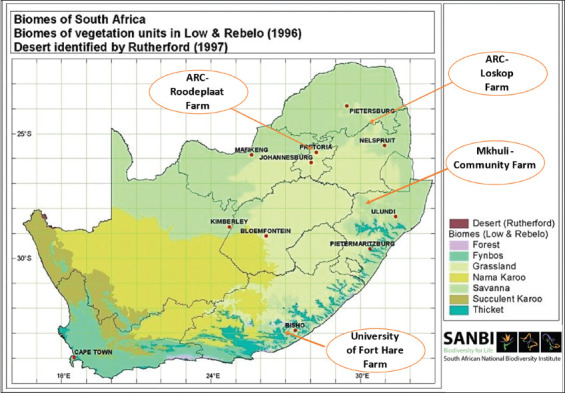
Location of the four experimental farms considered for tick counting. [Source: http://www.plantzafrica.com/vegetation/vegimages/biomes800.jpg].

Tick counts were conducted on 586 Nguni cattle over 2 years (May 2012–April 2014) from four locations: (1) Limpopo Province’s Loskop Research Farm of the Agricultural Research Council (n = 124), which has an average annual rainfall of 509 mm, and temperatures that range from 19°C in June to 29°C in January, with an average annual temperature of 26°C. It comprises tropical forest, dense bushes and shrubs in semidesert areas. (2) ARC-Roodeplaat Research Farm is located in Gauteng Province (n = 143). It has an average rainfall of 573 mm per annum, and an annual average temperature of 24°C, and ranging from 19°C in June to 27°C in December. It comprises of open savannah veld and bush and thornveld. (3) Mukhuthali Nguni Community Farm is located in the Kwa-Zulu Natal Province (n = 224). It has an average rainfall of 688 mm per annum, and temperatures that range from 19°C in June to 27°C in January, with an annual average temperature of 23°C. It comprises tall bush grass with mostly acacia trees. (4) The University of Fort Hare farm is located in Alice in the Eastern Cape Province (n = 95). It has an average rainfall of 480 mm per annum, and temperatures ranging from 20°C in June to 26°C in February, with an annual average temperature of 24°C. It comprises false thorn trees with some savannah vegetation type. These farms are in different agroclimatic zones [38] (Table-1). Figure-1 presents a map of the four farms. The cattle were exposed to natural tick infestation at all four farms. On all farms, tick counts and species identification were carried out each month. In addition, all cattle were spray dipped monthly with a flumethrin pour-on formulation “Drastic Deadline” immediately after sampling.

**Table-1:** Summary of the climatological and vegetation characteristics of the four farms used in the study.

Farm (Province)	Minimum and maximum monthly rainfall (Annual Rainfall)	Average daily temperature range	Vegetation
Loskop (Limpopo)	0 mm in June and 92 mm in November (506 mm)	19°C in June to 29°C in January and annual average of 26°C	Tropical forest, dense bush and shrubs to semi-desert areas[38]
Roodeplaat (Gauteng)	0 mm in June and 110 mm in January (573 mm)	19°C in June to 27°C in December and annual average of 24°C	Open savannah veld and bush and thornveld [38]
Mukhuthali (KwaZulu-Natal)	3 mm in June and 122 mm in December (688 mm)	19°C in June to 27°C in January and annual average of 23°C	Tall bush grass with mostly acacia trees [38]
Fort Hare (Eastern Cape)	8 mm in July and 56 mm in March (480 mm)	20°C in June to 26°C in February and annual average of 24°C	False thorn trees with some savannah vegetation type [38]

### Tick counting

Tick species were identified during counting at each location. Throughout the experiment, the data were gathered by the same team of trained technicians. Each person counted and identified the species of ticks on one half of the animal at a time, two people counting one animal at a time. Adult ticks were counted on each animal by counting and identifying tick species on different body parts, including the head (excluding the inside ears), ears (inside the ears), neck (including the gullet), back, legs, belly (including the udder or testicles), perineum and tail (including underneath the tail). Tick counts and species identification were performed on only adult ticks that are not difficult to distinguish. Ticks of the genera *Hyalomma* and *Rhipicephalus* were classified at genus and subgenus levels. For example, there are two *Boophilus* ticks in South Africa, and we classified them as *Rhipicephalus* (*Boophilus*) spp. and similar naming for *Hyalomma* spp. due to the two *Hyalomma* species being prevalent in South Africa.

### Statistical analysis

The generalized linear model procedure of the statistical analysis system was used to analyze the data [39]. The models fitted accounted for the effects of location (farm), sex, year (May 2012–April 2013 = Year 1 and May 2013–April 2014 = Year 2), either month or season (cool-dry, cool-wet, hot-dry, and hot-wet) and age of the animal as a covariate. The models fitted are described below:


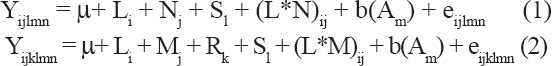


where: Y_ijklmn_ is the tick count;

µ is the overall mean;

L_i_ is the effect of the i^th^ location (i = Loskop, Roodeplaat, Mukhuthali, Fort Hare);

N_j_ is the effect of the j^th^ season (i = hot-dry, hot-wet, cool-wet, and cool-dry);

M_j_ is the effect of the j^th^ month (j = January to December);

R_k_ is the effect of the k^th^ year (k = 1, 2);

S_l_ is the effect of the l^th^ sex (l = Male, Female);

(L*M)_ij_ is the interaction effect of the i^th^ location and j^th^ month;

b is the partial regression coefficient of age on tick count;

A_m_ is the effect of the m^th^ age of the animals;

e_ijklmn_ are the random residuals.

## Results

The tick species observed were as follows: *A. hebraeum* (42%), *Rhipicephalus evertsi* (22%)*, Rhipicephalus* (*Boophilus*) spp. (16%), *Rhipicephalus appendiculatus* (11%), *Hyalomma marginatum* (5%), and *Rhipicephalus simus* (4%). *R. simus* was the least frequently encountered species and was found only at the University of Fort Hare farm. Table-2 summarizes the tick count data for the four locations. Loskop farm recorded the highest mean tick count of 30.69, the highest tick count per animal of 198, and the highest standard deviation of 19.79. In contrast, the highest tick load variation was observed on the Roodeplaat farm, followed by the Loskop farm and Fort Hare farm (Table-2).

**Table-2:** Summary statistics of tick counts data for four locations.

Location	Mean	SD	CV (%)	Min	Max
Loskop	30.69	19.79	64.48	0	198
Roodeplaat	25.97	18.66	71.85	0	118
Fort Hare	18.23	11.74	64.40	0	86
Mukhuthali	18.19	10.89	59.87	0	75

SD=Standard deviation, CV=Coefficient of variation, Min=Minimum tick count, Max=Maximum tick count. ^#^The mean and standard deviation were calculated from back transformed tick count data

### Species prevalence

**Figure-2:**
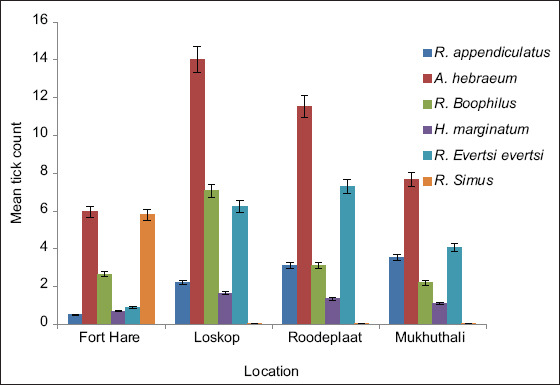
Tick loads for different species per location (farm).

The location had a significant effect on the total tick count per animal. The highest tick density per animal was found at the ARC Loskop Research Farm, followed by Roodeplaat, Mukhuthali, and Fort Hare. *A. hebraeum* and *Rhipicephalus* (*Boophilus*) spp. were the most prevalent species at Loskop farm (Figure-2). Between November and December 2012, an outbreak of heartwater disease was caused by the abundance of *A. hebraeum* ticks at Loskop farm. At Roodeplaat farm, *A. hebraeum* and *R. evertsi* had significantly greater counts than the other four species. At Mukhuthali Community farm, *A. hebraeum* had the highest count, followed by *Rhipicephalus evertsi evertsi* and *R. appendiculatus*. At Fort Hare farm, *A. hebraeum* and *R. simus* had greater (p < 0.05) counts than the other four species.

### Influence of climate on tick counts

**Figure-3:**
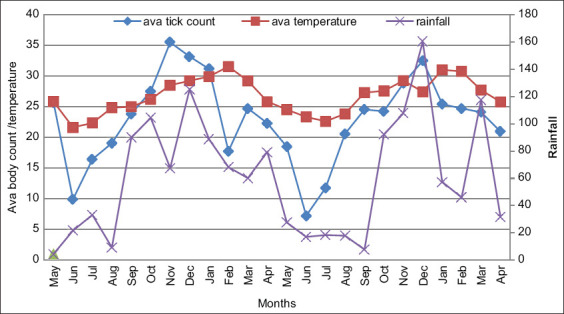
Association of monthly rainfall and average temperature (ava temperature) with average tick count on all body parts (ava tick count).

Figure-3 shows the distribution of average tick count, temperature, and rainfall over 2 years. The rainfall and tick count show the same distribution pattern throughout the study, indicating a relationship between the two.

**Figure-4:**
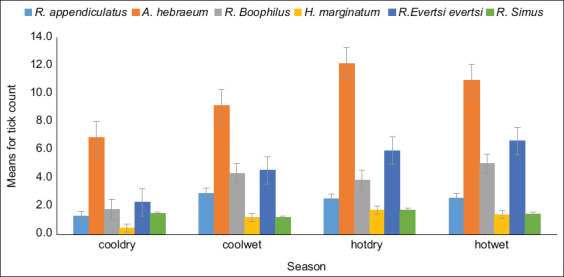
Seasonal variation in mean tick count by tick species over 2 years.

The season had a significant effect (p < 0.05) on the prevalence of tick species (Figure-4). Most species had the lowest count in the cool-dry season, which gradually increased over the cool-wet and hot-wet seasons and peaked in the hot-dry season. *A*. *hebraeum* ticks were prevalent in all seasons.

**Figure-5:**
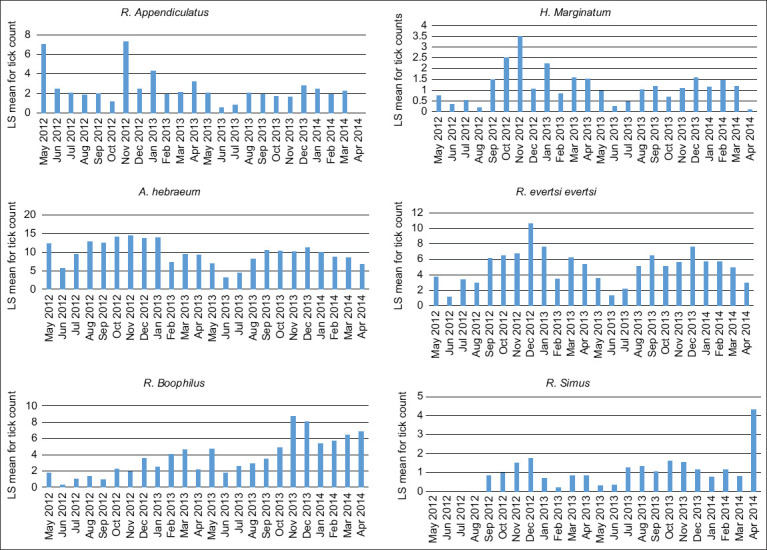
Monthly average tick counts per species over 2 years.

The number of ticks varied significantly by month and year. Years 1 and 2 were defined as May 2012–April 2013 and May 2013–April 2014, respectively. In Year 1, all species had the highest infestation in November and lowest infestation in June. In Year 2, tick infestations were highest in December and lowest in June. *A. hebraeum* tick load was highest between October and January in both years, whereas *R. evertsi evertsi* had the highest tick load in December. *Rhipicephalus* (*Boophilus*) ticks had low counts in Year 1 but much higher tick loads in Year 2. For 2 years of tick counting, each tick species’ monthly tick count patterns differ (Figure-5).

**Figure-6:**
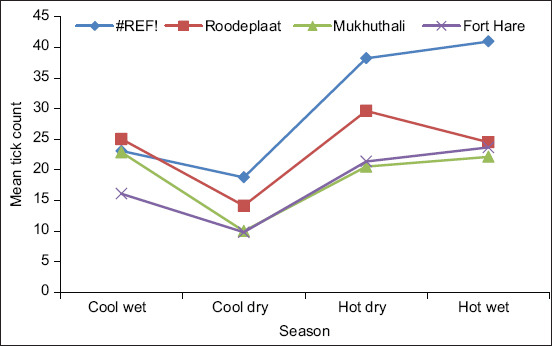
Interaction of location (farm) and season for mean tick count.

The interaction between location and season was significant (p < 0.001) for tick count (Figure-6). The magnitude and ranking of differences in tick counts among the farms varied from season to season. For instance, Loskop farm had the highest tick count throughout the year, while Roodeplaat farm had the highest count during the cool seasons.

### Tick distribution on the host body

**Figure-7:**
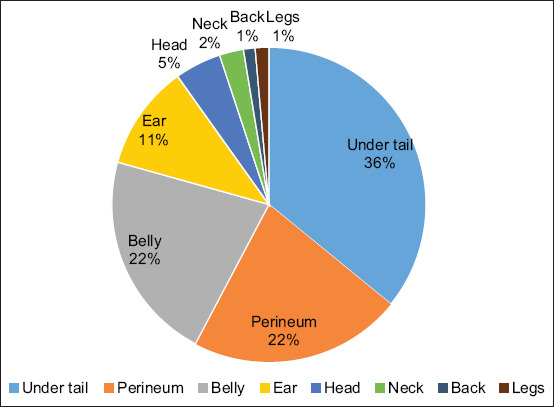
Percentage of total tick count per body location of the animal.

**Figure-8:**
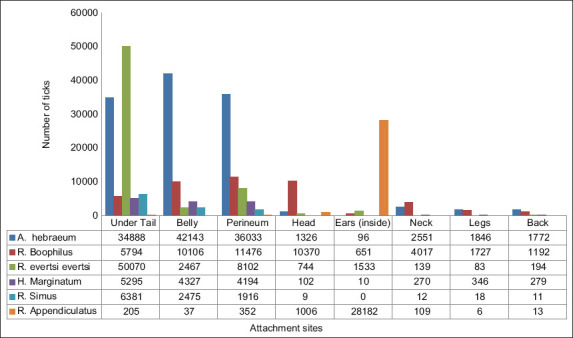
Distribution of total tick count per species on different body locations.

Figure-7 shows the distribution of ticks on various body parts. The preferred sites for attachment were the belly, perineum and under the tail. The undertail region had the greatest infestation (36%), followed by the perineum (22%), belly (22%) and inside the ear (11%). Less than 10% of the infestation was found in other body parts. *A. hebraeum* was located across the body, with the highest occurrence on the belly. The highest counts for *R. evertsi evertsi* were obtained under the tail, while *R. appendiculatus* were most prevalent inside the ears. Figure-8 shows the distribution of all ticks by species on various body parts. Under the tail, *R. evertsi evertsi* (49%) was the most prevalent, followed by *A. hebraeum* (34%). On the belly, 69% of the ticks were *A. hebraeum* followed by 16% of *Rhipicephalus* (*Boophilus*) spp. In the perineal region, 58% were *A. hebraeum* followed by 18% of *Rhipicephalus* (*Boophilus*) spp. In the ear, 93% of ticks found were *R. appendiculatus*. On the head, 77% of the ticks found were *Rhipicephalus (Boophilus)* spp. Thus, most tick species tended to have a preferred site for attachment to the host.

**Figure-9:**
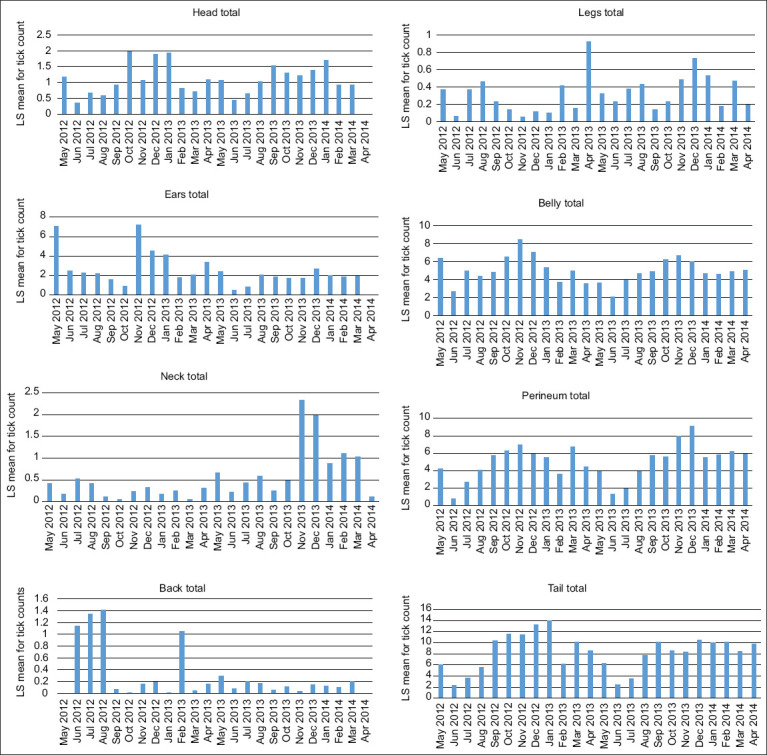
Least squares mean for monthly tick count on different body locations.

Figure-9 presents tick distribution patterns on the different body locations in each month. For years 1 and 2, highest tick loads on the belly were seen in November, whereas the lowest tick loads were seen in June. The belly, perineum and tail had similar distribution patterns for the observed years.

## Discussion

The four locations included in the present study show significant area variation in the distribution of tick species that infest cattle. The species observed were as follows: *A. hebraeum*, *R. evertsi evertsi*, *Rhipicephalus* (*Boophilus*) spp., *R. appendiculatus*, *H. marginatum*, and *R. simus*. These were the same species identified by Katiyatiya *et al*. [19] and Marufu *et al*. [37] in the Eastern Cape Province of South Africa and by Assefa, Tessema and Gashaw, Tiki, and Addis [40-42] in Ethiopia. *R. simus* was observed only at the University Fort Hare farm in the Eastern Cape Province, and its recent infestations in cattle were also reported by Katiyatiya *et al*. [19] and Nyangiwe *et al*. [27]. In the past, this tick species was known to infest dogs, cats, cheetahs, lions, sheep and goats and was primarily found in moist areas [43, 44]. The occurrence of *R. simus* at Fort Hare in the present study might be because this farm is located in a moist region of the Eastern Cape and has mixed grazing practices with small stock.

*A. hebraeum* was the most prevalent and widely distributed tick species in this study. Similar observations were recorded in Limpopo Province of South Africa [22]. This tick is usually found in warm and hot, dry, harsh areas and occurs where cattle dominate as the most important domestic host [45]. The abundance of *A. hebraeum* was also observed in Southern African buffalos [44, 46]. Due to the long mouthpart of this species, which damages these commodities and lowers their value on the global market, heavy infestation of this species results in losses in cattle production and damage to hides and skins [47]. Mapiye *et al*. [48] reported that the value of Nguni hides drops due to tick bite damage and decreases the quality of skin hide products in South Africa. During the present study, the *A. hebraeum* tick burden at Loskop farm resulted in an outbreak of heartwater between November and December 2012. Heartwater was observed only in the Angus × Nguni cross and resulted in 25% mortality of this crossbreed on the Loskop farm (data unpublished). However, there was no incidence of heartwater mortality in Nguni cattle used in this study. Over time, tick-borne disease mortality was noted, and studies on host resistance to tick infestation in South African cattle [31, 35].

*R. evertsi evertsi* was the second most abundant tick species. The abundance of this tick species was previously recorded in the Eastern Cape Province of South Africa [10, 12, 49] and various regions of Ethiopia [41, 42]. *R. evertsi evertsi* was also reported as Africa’s most prevalent species in the genus *Rhipicephalus* [44]. This species is known to be found mostly in the savannah areas. Its other target hosts are zebra and small ruminants [40], and it is found in all year seasons [27]. *R. evertsi* is a vector of *Anaplasma marginale*, which causes anaplasmosis and causes considerable economic loss to dairy and beef industries worldwide.

*Rhipicephalus* (*Boophilus*) spp., including *R. microplus* and *R. decoloratus*, were the third most abundant tick species. *R. microplus* was reported as the source of *R. decoloratus* displacement in the Eastern Cape Province of South Africa by Nyangiwe *et al*. [12]. The distribution of *R. decoloratus* ticks was reported in South Africa [12, 22, 50] and in other African countries [40, 51], and *R. microplus* abundance was reported in Brazil [5, 52] and Malaysia [53]. The resistance of *R. decoloratus* to acaricide was observed in Adehan *et al*. [54], Kariuki *et al*. [55]. This tick species transmit *Babesia* *bigemina* and *A. marginale* to cattle, and heavy infestations can cause tick worry and anemia [56]. The brown ear tick, also known as *R. appendiculatus*, was the fourth most prevalent species of ticks and preferred the ear’s interior (93%) for attachment. Severe infestations of *R. appendiculatus* were observed to cause substantial damage to animals’ ears. *H. marginatum* and *R. simus* were the least prevalent species.

The differences in the tick load among the four locations may be attributable to differences in latitude and hence average daily temperatures. The Loskop and Roodeplaat farms are more toward the northern part of South Africa, which is warmer. In contrast, Mukhuthali Community and Fort Hare farms are close to the cooler south coastal region. The higher tick count at Loskop compared with the other farms is likely caused by higher temperatures. The variation in vegetation may partly explain the differences in the tick loads among the four farms. In this study, the main biomes observed in all four farms were savannah and grassland, which are often associated with increased tick loads [57]. Scholtz *et al*. [1] noted that the Loskop research farm is an endemic tick area because its forest trees, tall grass, and hot, dry climatic conditions attract ticks.

Compared to the cool-wet and dry seasons, higher tick loads were seen during the hot-dry and hot-wet seasons. Similarly, several studies [31, 32, 58, 59] reported a higher tick load in the hot-wet season when comparing different breeds of cattle in South Africa. In Brazil, hot and rainy seasons are associated with higher tick infestation [5, 52]. This is probably because humid, hot weather encourages the growth and survival of ticks. Tick proliferation is normally enhanced when there are high temperatures and humidity [60, 61]. In addition, the magnitude and ranking of differences in tick count among the different farms were inconsistent across the seasons. Therefore, the interaction most likely develops due to Loskop’s tick count being comparable to other locations during the cool-wet season and significantly higher than other locations during the hot-wet season. The decrease, relative to the prior hot-dry season, in tick numbers at Roodeplaat observed during the hot-wet season might have contributed to the significance of the interaction. The constant to slightly rising tick counts at the other three locations are evidence of this.

The observed significant effect of age of animals on tick counts agrees with the report of Maruf*u et al*. [37], who also observed lower ticks in younger animals than in older ones. The age effect is attributed to some form of innate protection that declines with age [62].

There seems to be a preferred location for tick attachment on the host body. Ticks were also discovered under the tail and then in the perineum and belly. *A. hebraeum* were in most body locations, with the highest occurrence in the belly. The highest counts for *R. evertsi evertsi* were obtained under the tail, whereas *R. appendiculatus* were most prevalent inside the ears. The higher infestations under the tail could be because ticks prefer warm, moist, hidden sites with a good vascular supply and thin skin, and there is also thought to be a pleasing effect of the anal odors on ticks [10, 32]. The external genitals and inguinal/groin regions of the body are highly supplied with blood [40]. Ticks prefer to attach to body parts with short hair and softer or thinner skin for their mouth parts to easily enter vascularly dense areas for feeding [32, 63].

Environmental factors such as location, month, and season influence tick infestation. Tick prevalence was generally high and a major challenge in these four locations. Several studies showed the same challenge in the Eastern Cape Province of South Africa [19, 27, 32], while Scholtz *et al*. [1] raised the same concern in the Loskop area. Acaricides are used during the hot-dry and hot-wet seasons because of their high tick loads to avoid significant losses in cattle production. However, there is a need to develop sustainable and more cost-effective strategies for tick control that can counter the effects of global warming or tick burden in livestock production. A viable option is an integrated strategy incorporating genetic improvement using traditional or marker-assisted selection techniques. Therefore, it is necessary to determine the extent to which the large variation in tick count observed in the present study is under genetic control.

## Conclusion

All four experimental farms had the same prevalence of tick species, excluding *R. simus*. The tick load in Nguni cattle varies according to these agroecological zones, with warmer locations tending to have higher tick loads and *A. hebraeum* being the most prevalent and widely distributed species. The year, season, month of counting, and animal age also influenced tick loads. Tick count was significantly higher in the hot-dry and hot-wet seasons than in cool-wet and cool-dry seasons. There were within-breed variations in tick counts and adaptation of animals to different environmental conditions.

## Authors’ Contributions

NOM: Study conception and design, analysis and interpretation of results, and drafted the manuscript. CB: Data analysis and revised the manuscript. KD: Study conception and supervision and revised the manuscript OM and VR: Data analysis and revised the manuscript. NN: Data collection and revised the manuscript. AM: Supervised the study and revised the manuscript. All authors have read and approved the final manuscript.
